# English teachers’ perceptions of AI-supported writing instruction: implications for professional development

**DOI:** 10.3389/fpsyg.2026.1806618

**Published:** 2026-04-10

**Authors:** Mengmeng Wu, Jinfang Yao, Hisham Noori Hussain Al-Hashimy

**Affiliations:** 1 Department of General Education, Henan Vocational University of Science and Technology, Zhoukou, Henan, China; 2 School of Business, Huanghe University of Science and Technology, Zhengzhou, Henan, China; 3 School of Management, Universiti Sains Malaysia, George Town, Penang, Malaysia

**Keywords:** artificial intelligence in language education, instructional agency, pedagogical integration, professional development, teacher cognition

## Abstract

The increasing adoption of artificial intelligence (AI) in English writing instruction has drawn growing attention to teachers’ perceptions of AI-supported writing instruction (TPAI) in classroom contexts. However, existing research has predominantly focused on technological affordances and student outcomes, offering limited insight into teachers’ instructional agency (IA) and professional support needs. This study aims to examine how English TPAI influences the pedagogical integration of AI-supported writing instruction (PIAI), with particular emphasis on the roles of IA and professional development (PD), as well as the moderating effect of institutional and ethical support (IES). A quantitative research design was employed using survey data collected from English teachers in formal educational institutions. Partial least squares structural equation modeling (PLS-SEM) was applied to test the hypothesized relationships among TPAI, IA, PD, IES, and PIAI. The results show that TPAI significantly enhances IA (*β* = 0.40, *p* < 0.01) and PD engagement (*β* = 0.65, *p* < 0.01). Both IA (*β* = 0.19, *p* < 0.05) and PD (*β* = 0.68, *p* < 0.01) positively influence PIAI, explaining 57% of the variance. IES significantly moderates the relationship between IA and PIAI (*β* = −0.18, *p* < 0.05). These findings underscore the importance of targeted PD and coherent IES structures in facilitating effective PIAI, and provide a teacher-centered perspective on how teacher preparedness and institutional context jointly shape the early adoption of AI in writing instruction.

## 1 Introduction

The rapid advancement of artificial intelligence is reshaping educational practices across disciplines worldwide (Kamalov et al., 2023). In recent years, AI-based technologies have been increasingly integrated into teaching and learning environments to support instructional design, assessment, and feedback processes (Yildiz Durak and Onan, 2025). Within language education, AI has gained particular prominence in writing instruction, where tools such as automated feedback systems, grammar checkers, and generative language models are now commonly used to support learners throughout the writing process (Alharbi, 2023). These developments have prompted growing interest in how AI can enhance traditional writing instruction by providing timely feedback, individualized support, and opportunities for learner autonomy. Traditionally, writing instruction in English language education has relied heavily on teacher-generated feedback, peer review, and iterative drafting processes (Tran, 2025). While these approaches remain pedagogically valuable, they are often constrained by time, workload, and class size, limiting the frequency and depth of feedback teachers can provide (Radovan and Radovan, 2024). AI-supported writing instruction has been proposed as a means of complementing traditional practices by offering immediate linguistic feedback and supporting revision, thereby allowing teachers to focus more on higher-order aspects of writing, such as organisation, argumentation, and coherence (Guo et al., 2025). Despite these potential benefits, the effective use of AI in writing instruction depends not only on technological capability but also on teachers’ professional judgement and instructional practices.

Existing research on AI in education has expanded rapidly, with studies largely examining student performance, tool effectiveness, or ethical concerns related to AI use (Phua et al., 2025). However, relatively little attention has been paid to teachers’ perceptions of AI-supported writing instruction (TPAI), particularly regarding how these perceptions influence instructional decision-making and professional practice. Teachers play a central role in mediating the pedagogical use of AI, yet their beliefs, confidence, and readiness to integrate AI tools remain under-examined. This represents a significant theoretical gap, as teacher cognition research has long established that instructional practices are shaped by teachers’ beliefs and perceptions rather than by instructional tools alone (Qiu, 2025). Moreover, the global evolution of AI integration in education has been uneven. Much of the existing empirical evidence originates from well-resourced educational contexts in developed countries, where institutional infrastructure and professional support for AI adoption are more readily available (Reimers et al., n.d.). In contrast, developing educational contexts remain underrepresented in the literature, despite distinct challenges related to policy guidance, professional development (PD), and the ethical governance of AI use. Understanding how teachers in such contexts perceive and engage with AI-supported writing instruction is, therefore, essential for developing inclusive and context-sensitive theoretical models and PD strategies. In addition to this contextual gap, the literature has yet to sufficiently theorize the mechanisms through which TPAI translates into classroom practice. While positive attitudes toward educational technologies are often assumed to lead to adoption, research suggests that teachers’ confidence in instructional decision-making, commonly referred to as instructional agency (IA), plays a critical role in shaping how technologies are enacted in practice (Dexter, 2023). Furthermore, PD has been identified as a key factor in supporting teachers’ engagement with instructional innovation. However, it is frequently treated as a background condition rather than a central explanatory mechanism in studies of AI integration (Tammets and Ley, 2023).

Against this background, the present study seeks to address both theoretical and practical gaps by examining English TPAI and exploring the implications for PD. Specifically, the study investigates how TPAI influences IA and the PIAI in writing instruction and examines the role of PD in supporting this process. By adopting a teacher-centred perspective, the study responds to calls for more theoretically grounded research on AI integration that accounts for teachers’ professional judgement and learning needs. Accordingly, the study is guided by the following objectives:

1. To examine English TPAI;
2. To investigate the relationship between TPAI and IA;
3. To explore how IA influences the PIAI;
4. To examine the mediating role of PD in these relationships.

Based on the conceptual framework and the reviewed literature, the study proposes a set of hypotheses that explain how TPAIs are translated into instructional practice and highlight the central role of PD in supporting effective and ethical AI integration. As shown in Table 1, prior research has largely examined isolated aspects of AI integration in education. Few studies have empirically modeled the combined roles of TPAI, IA, PD, and IES in explaining PIAI, particularly in the context of English writing instruction. This study addresses this gap using a theory-driven PLS-SEM approach.

**Table 1:** Empirical evidence on AI integration, teacher cognition, and professional development in education.

Authors	Context	Teachers’ perceptions	Instructional agency	Professional development	Institutional/ethical support	Pedagogical integration focus	Research design
Torres (2022)	Technology-mediated language learning	Yes	Limited	No	No	Classroom technology use	Conceptual
Bower et al. (2024)	AI in education	Yes	No	Limited	No	Instructional effectiveness	Survey
Li et al. (2025)	AI-supported teaching	Yes	No	Yes	Limited	Teaching innovation	Review
Stevens (2025)	Teacher professional learning	Yes	Yes	Yes	No	Digital pedagogy	Qualitative
Salas-Pilco et al. (2022)	AI tools in higher education	Limited	No	No	Yes	Learning analytics	Review
Wilson (2023)	Educational technology adoption	Yes	Limited	Yes	No	Technology integration	Meta-analysis
Ayanwale et al. (2022)	Teacher AI readiness	Yes	Yes	Limited	No	Classroom implementation	Survey
Holmes and Porayska-Pomsta (2023)	Ethics of AI in education	No	No	No	Yes	Governance and ethics	Conceptual
This study	English writing instruction	Yes	Yes	Yes	Yes	Pedagogical integration of AI-supported writing	PLS-SEM

Source(s): authors own work.

For clarity, the main constructs examined in this study are defined as follows: Teachers’ Perceptions of AI (TPAI) refer to teachers’ beliefs about the usefulness and pedagogical relevance of AI tools. Instructional Agency (IA) refers to teachers’ perceived professional capacity to make instructional decisions regarding AI use. Professional Development (PD) refers to teachers’ learning opportunities related to AI-supported instruction. Institutional and Ethical Supports (IES) are contextual supports that guide responsible AI use. Pedagogical Integration of AI (PIAI) refers to teachers’ reported use of AI to support writing instruction. These abbreviations are used throughout the paper for brevity.

## 2 Literature review and hypotheses development

### 2.1 Theoretical perspectives on AI-supported writing instruction

The growing presence of artificial intelligence in educational settings has prompted renewed attention to how emerging technologies shape teaching practices (Dimitriadou and Lanitis, 2023). In English language education, AI-supported writing instruction refers to the use of intelligent systems, such as automated feedback tools and generative language models, to support learners during the writing process (Liu et al., 2025). Understanding teachers’ engagement with such tools requires a theoretically grounded perspective that recognizes teaching as a cognitively and socially situated activity (Poquet, 2024). Teacher cognition theory provides a useful foundation for examining how teachers interpret and respond to instructional innovations. According to this perspective, teachers’ beliefs, perceptions, and prior experiences strongly influence classroom decision-making and instructional behavior (Bakshi, 2023). The introduction of AI into writing instruction, therefore, does not lead to uniform pedagogical change. However, it is mediated by TPAI of the instructional value, risks, and legitimacy of AI tools. In addition, the Technology Acceptance Model suggests that individuals’ adoption of new technologies is influenced by perceived usefulness and perceived ease of use (Liesa-Orús et al., 2023). Within educational contexts, these perceptions are closely linked to pedagogical beliefs and professional identity (Suarez and McGrath, 2022). Complementing these perspectives, Technological Pedagogical Content Knowledge theory emphasises that meaningful technology integration depends on teachers’ ability to align technological knowledge with pedagogical strategies and subject matter expertise (Zhang and Li, 2025). Although these theories have been widely applied in studies of educational technology, existing research on AI-supported writing instruction has primarily focused on student outcomes or tool performance, often overlooking the cognitive and professional processes through which teachers enact AI in practice. This indicates a theoretical gap regarding the mechanisms linking TPAI to instructional decision-making in writing pedagogy. Addressing this gap is essential for advancing theory in AI-enhanced language education. Therefore, this study proposes an integrated framework explaining how TPAI, PD, IA, and IES jointly shape PIAI.

### 2.2 Teachers’ perceptions of AI-supported writing instruction (TPAI) and instructional agency (IA)

IA refers to teachers’ perceived capacity to exercise professional judgement, make autonomous pedagogical decisions, and adapt instructional practices to contextual demands (Mohammad Nezhad and Stolz, 2025). From the perspective of teacher cognition theory, teachers’ beliefs about instructional tools shape not only their willingness to adopt innovations but also their sense of control over pedagogical processes. In the context of AI-supported writing instruction, teachers who perceive AI tools as useful for enhancing feedback quality, supporting revision, or managing instructional workload are more likely to view these tools as resources that extend, rather than diminish, their professional role. Research on the Technology Acceptance Model supports this view, demonstrating that positive perceptions of usefulness strengthen engagement with instructional technologies (An et al., 2022). Conversely, concerns about academic integrity, ethical accountability, and the loss of pedagogical authority may reduce teachers’ sense of IA, leading to cautious or minimal use of AI tools. While prior studies have documented mixed teacher attitudes toward educational technologies, few have explicitly conceptualized IA as the mechanism through which perceptions influence pedagogical engagement with AI. This study addresses this limitation by positioning IA as a central explanatory construct.

*H1: Positive perceptions of AI-supported writing instruction are positively associated with English teachers’ instructional agency (IA) in writing pedagogy.*

The rationale for this hypothesis is grounded in teacher cognition theory and the Technology Acceptance Model, which together suggest that favorable evaluations of instructional technologies strengthen teachers’ confidence and capacity to act pedagogically.

### 2.3 Instructional agency (IA) and pedagogical integration of AI-supported writing instruction (PIAI)

Pedagogical integration refers to the extent to which AI tools are meaningfully incorporated into instructional design, classroom interaction, and assessment practices. According to the Technological Pedagogical Content Knowledge theory, effective integration requires teachers to make informed pedagogical decisions that align technology use with disciplinary goals and learning outcomes (Zhang and Li, 2025). Teachers with a strong sense of IA are more likely to engage critically with AI-supported writing tools (Li and Wilson, 2025). Such teachers may combine AI-generated feedback with human judgment, design writing tasks that promote reflection rather than automation, and guide students in evaluating the limitations of AI output. In contrast, limited IA may lead to procedural or compliance-oriented use of AI tools that provide little pedagogical benefit. Existing research on technology integration has often assumed a direct relationship between access to digital tools and instructional use, underestimating the agentic role of teachers in shaping classroom practice. By foregrounding IA, this study contributes a more theoretically robust explanation of how AI-supported writing instruction is enacted in practice.

*H2: Instructional agency (IA) positively affects the pedagogical integration of AI-supported writing instruction (PIAI).*

This hypothesis is grounded in TPACK theory and research on teachers’ IA, which together emphasize that professional autonomy is a prerequisite for meaningful technology integration. It is important to note that IA is not shaped solely by teachers’ individual perceptions or competencies, but also by contextual conditions such as institutional policies, technological infrastructure, leadership expectations, and ethical guidelines governing AI use (Mouta et al., 2025). Prior research suggests that IA develops through the interaction between individual capacity and structural conditions rather than through individual cognition alone (Sang, 2022). In this study, this contextual dimension is reflected through IES, which represent environmental conditions that may either enable or constrain how teachers exercise their instructional judgement when integrating AI into writing instruction.

### 2.4 Professional development (PD) as a mediating mechanism

PD plays a central role in supporting teachers as they respond to instructional innovation. Situated learning theory conceptualizes PD as a social process through which teachers construct knowledge, negotiate professional norms, and develop shared practices within communities of practice (Dlamini et al., 2024). In relation to AI-supported writing instruction, PD can enhance teachers’ technological understanding, pedagogical strategies, and ethical awareness. More importantly, it may help translate TPAI into actionable instructional capacity by strengthening teachers’ preparedness, perceived legitimacy of AI use, and professional capability (Chiu et al., 2025). Without adequate PD, positive perceptions of AI may not translate into meaningful instructional change. Although PD is frequently discussed in the literature, it is often treated as a descriptive variable rather than a theoretically grounded mechanism. This study addresses this gap by conceptualizing PD as a mediating process that explains how TPAI influences IA. Although this hypothesis focuses on the relationship between TPAI and IA, PD may also contribute to teachers’ ability to translate their perceptions into practical instructional implementation. Therefore, in addition to its conceptual role in strengthening IA, PD may indirectly support the PIAI by enhancing instructional competence. This interpretation is consistent with prior research suggesting that PD often supports both teachers’ professional judgement and their capacity for instructional implementation.

*H3: Professional development (PD) mediates the relationship between teachers’ perceptions of AI-supported writing instruction (TPAI) and their instructional agency (IA).*

This hypothesis is theoretically anchored in situated learning theory, which emphasises the role of social learning contexts in shaping professional practice.

### 2.5 Institutional and ethical support (IES) as a moderating condition

Institutional theory highlights the influence of organisational norms, policies, and regulatory frameworks on how innovations are adopted and legitimized (Balzano et al., 2025). In educational contexts, clear institutional guidance on the ethical use of AI can shape teachers’ confidence and willingness to integrate AI tools into instruction. When IES is well established, teachers may feel more secure in exercising IA and experimenting with AI-supported writing practices. In contrast, ambiguous or weak institutional policies may constrain PIAI, even among confident, well-trained teachers. This suggests that the relationship between IA and PIAI is contingent on broader organisational conditions.

*H4: Institutional and ethical support (IES) positively moderates the relationship between instructional agency (IA) and the pedagogical integration of AI-supported writing instruction (PIAI).*

This hypothesis is grounded in institutional theory, which emphasises that organisational contexts shape the enactment of individual professional capacity. To clarify the conceptual structure of this study, professional development is positioned as the primary developmental mechanism through which teachers translate their perceptions of AI-supported writing instruction into pedagogical practice. At the same time, IA represents the individual capacity that enables teachers to exercise professional judgement in AI integration. In this framework, TPAI serve as an initial cognitive driver, PD functions as the capability-building process, IA reflects teachers’ professional decision-making capacity, and the pedagogical integration of AI (PIAI) represents the observable instructional outcome. Institutional and ethical supports (IES) are treated as contextual conditions that may strengthen or compensate for differences in teachers’ IA. This structure allows the study to integrate TPAI, PD, and IES context into a single explanatory model without altering their distinct theoretical roles. These supports can be understood as contextual conditions that shape how IA can be enacted in practice.

### 2.6 Research model

**Figure 1:**
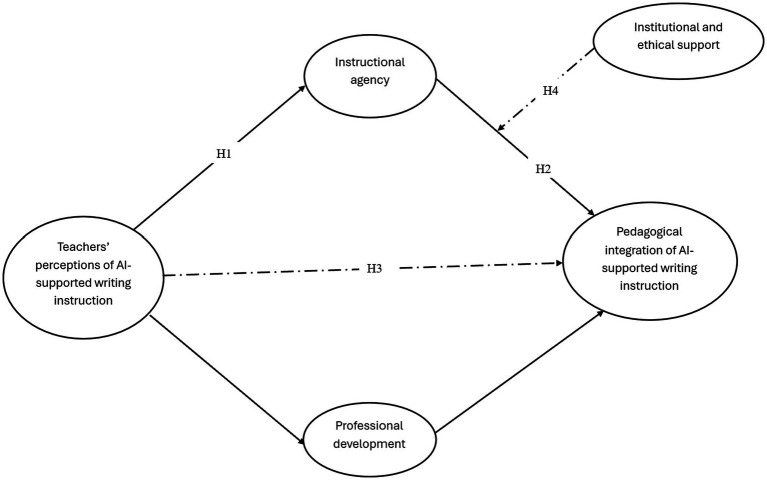
Conceptual framework of the study, showing the various hypotheses. Source(s): author’s own work.

The research model presented in Figure 1 was developed based on the theoretical insights derived from the literature review. The model identifies TPAI as the primary independent variable and the PIAI as the dependent variable. IA is positioned as a central mediating variable that explains how TPAIs are translated into instructional practice. In addition, PD is included as a complementary mediating mechanism that supports teachers in converting their perceptions into effective PIAI. IES is incorporated as a moderating variable that influences the strength of the relationship between IA and PIAI. Collectively, the model captures both the direct and indirect pathways through which TPAI shapes the integration of AI-supported writing instruction within educational contexts.

### 2.7 Writing instruction in AI-supported environments

Writing instruction represents a distinctive pedagogical domain because writing involves complex cognitive, metacognitive, and revision processes rather than simple knowledge reproduction. According to the cognitive process theory of writing, writing involves recursive processes of planning, translating ideas into text, and reviewing or revising (Silva, 2022). These processes require instructional support to help students organize ideas, develop arguments, and improve the quality of their texts through feedback and revision. AI-supported writing tools are particularly relevant in this instructional context because they can assist with idea generation, language development, automated feedback, and revision suggestions. These affordances align with process-oriented writing instruction, which emphasizes drafting, feedback, and continuous improvement rather than only final written products (Cheung, 2023). As a result, teachers must exercise professional judgement in determining how AI tools should support learning without reducing students’ independent writing development. Furthermore, writing instruction frequently emphasizes self-regulated learning, including goal setting, monitoring progress, and making revision decisions (Jansen et al., 2024). AI tools may influence these processes by providing automated suggestions and feedback, which increases the importance of teachers’ IA in deciding how and when such tools should be used pedagogically. From this perspective, examining TPAI, PD, and IA is particularly relevant in writing contexts because AI integration directly affects how students plan, draft, and revise their work. By grounding AI integration within writing pedagogy, this study positions PIAI not simply as technology adoption but as a professional instructional decision concerning feedback practices, student authorship, and the development of writing competence.

## 3 Methodology

### 3.1 Research design and analytical approach

The study employed a quantitative, cross-sectional research design to investigate the relationships among English TPAI, IA, PD, IES, and the PIAI. A quantitative approach was appropriate because the research aimed to test theoretically grounded relationships and to examine direct, mediating, and moderating effects within a structured conceptual framework, rather than to explore individual experiences in depth (Moschis, 2024). Partial least squares structural equation modeling (PLS-SEM) was adopted as the primary analytical technique. This approach is well-suited to theory-driven research that prioritizes explanation and prediction by examining mediation and moderation mechanisms (Anwar et al., 2025). In addition, the proposed model includes formative constructs, which are more appropriately analysed using PLS-SEM than covariance-based structural equation modeling (Hair and Alamer, 2022). PLS-SEM is also suitable for complex models estimated with survey data and does not impose strict distributional assumptions, making it appropriate for educational research contexts (Batra, 2025).

### 3.2 Instrument development and data collection procedures

Data were collected using a structured questionnaire designed to measure the constructs specified in the research model. The questionnaire comprised five sections. The first section collected demographic information, including teaching experience, institutional level, and prior exposure to AI-supported instructional tools. The remaining sections measured TPAI, IA, PD, IES, and the PIAI. Measurement items were adapted from established instruments reported in prior research on teacher cognition, educational technology adoption, IA and PD (Richter and Richter, 2024). All items were revised to ensure relevance to the context of English writing instruction and contemporary AI-supported pedagogical practices. To establish content validity, the initial questionnaire was reviewed by six experts with backgrounds in English language education, educational technology, and quantitative research methodology. Based on their feedback, minor revisions were made to improve clarity and contextual alignment. A pilot study was subsequently conducted with 36 English teachers whose professional characteristics were comparable to those of the target population. The pilot study confirmed that the questionnaire items were clear, comprehensible, and suitable for large-scale data collection. All the items were identified through prior research and confirmed by experts in English language education, educational technology, and quantitative research methodology. Table 2 presents the constructs with their respective sources.

**Table 2:** Study constructs and measurement items.

Construct	Item code	Questionnaire item	Source
Teachers’ perceptions of AI-supported writing instruction (TPAI)	TPAI1	AI-supported tools can enhance the quality of students’ writing.	Can (2025)
	TPAI2	AI-supported writing tools are pedagogically useful for English writing instruction.	
	TPAI3	The use of AI in writing instruction aligns with my teaching objectives.	
	TPAI4	I feel confident about the educational value of AI-supported writing tools.	
Instructional agency (IA)	IA1	I feel autonomous in deciding when to use AI-supported tools in writing instruction.	Li et al. (2023)
	IA2	I can exercise professional judgement when integrating AI into writing lessons.	
	IA3	I am confident in adapting AI-supported tools to suit my instructional needs.	
	IA4	I feel responsible for decisions related to AI use in my writing classes.	
Professional development (PD)	PD1	I have received adequate training on using AI-supported tools for writing instruction.	Tan et al. (2025)
	PD2	PD activities have improved my ability to use AI in writing instruction.	
	PD3	Training opportunities address both pedagogical and ethical aspects of AI use.	
	PD4	Ongoing professional learning supports my effective use of AI in writing instruction.	
Institutional and ethical support (IES)	IES1	My institution provides clear guidelines for the ethical use of AI in writing instruction.	Drolet et al. (2023)
	IES2	Institutional policies support the responsible integration of AI in English teaching.	
	IES3	I receive adequate organisational support when implementing AI-supported writing instruction.	
	IES4	My institution clearly communicates ethical considerations related to AI use.	
Pedagogical integration of AI-supported writing instruction (PIAI)	PIAI1	I integrate AI-supported tools into writing instruction in a purposeful manner.	Engeness (2025)
	PIAI2	AI-supported tools are embedded into my writing lesson design.	
	PIAI3	I use AI tools to support feedback and revision in writing tasks.	
	PIAI4	AI-supported writing tools align with the learning outcomes in my classes.	
	PIAI5	AI use enhances students’ engagement with writing activities.	

Source(s): authors own work.

### 3.3 Measurement model specification and justification of formative constructs

All constructs in the study were specified as formative. This specification was retained because the indicators were conceptualized as distinct components that jointly define each construct rather than as interchangeable manifestations of a single latent trait. For example, PD in this study includes training adequacy, improvement in instructional ability, attention to ethical issues, and ongoing learning support; these dimensions contribute different aspects of the construct and do not need to covary at the same level to remain conceptually meaningful. Similarly, IA is represented by autonomy, judgement, adaptation, and responsibility, which together form the construct but are not assumed to be redundant reflections of a single underlying response tendency. From this perspective, changes in one indicator do not necessarily imply proportional changes in the others, which supports formative specification. This decision was guided by conceptual and methodological considerations rather than convention. TPAI, IA, PD, and PIAI are multidimensional concepts, shaped by distinct indicators that are not interchangeable and are not assumed to covary strongly. In formative measurement models, indicators are conceptualized as causes of the construct rather than reflections of an underlying latent variable (Kock, 2025). For example, PD encompasses multiple dimensions such as pedagogical training, technical competence, and ethical awareness, each of which contributes uniquely to the construct. Similarly, PIAI reflects a range of instructional practices that collectively represent effective AI-supported writing instruction. Modeling these constructs reflectively would risk construct misspecification. This approach aligns with established methodological guidelines for formative measurement models (Aguirre-Urreta et al., 2024). To support formative construct validity, redundancy analysis was conducted using a global item for each construct, in accordance with recommended practice in structural equation modeling research (Cheung et al., 2024). In addition to indicator weights and multicollinearity diagnostics, redundancy analysis was used to assess the convergent validity of the formative constructs by comparing each formative specification with a corresponding global item representing the overall construct (Cheung et al., 2024). This procedure follows recommended practice for formative measurement models and provides supplementary evidence that the indicator sets adequately capture the intended construct domain (Cheung et al., 2024). The redundancy analysis indicated acceptable convergent validity according to recommended thresholds, providing additional empirical support for the adequacy of the formative construct specification (Cheung et al., 2024). Because AI adoption in education was still emerging at the time of data collection, the questionnaire items were designed to capture TPAI, IA, PD and IES rather than only their current level of implementation. This approach allows examination of instructional readiness and professional judgement even among teachers with limited direct experience, which is common in studies of emerging educational technologies.

### 3.4 Measurement scales and assessment of reliability and multicollinearity

TPAI, IA, PD, and IES were measured on five-point Likert-type scales ranging from strongly disagree to agree strongly. The PIAI was measured using a seven-point Likert-type scale. The use of different scale formats was intended to reduce response pattern bias and to minimize the likelihood of common method bias (CMB) (Podsakoff et al., 2024). As the constructs were modeled formatively, internal consistency reliability measures such as Cronbach’s alpha, composite reliability, and average variance extracted were not applied, as these indices are not appropriate for formative constructs (Cheung et al., 2024). Instead, multicollinearity among indicators was assessed using variance inflation factor values.

### 3.5 Sampling strategy and sample size adequacy

The target population comprised English-language teachers working in formal educational institutions in China, including secondary schools, colleges, and universities, across different levels of the national education system. Inclusion criteria required that participants have at least a basic familiarity with AI-supported instructional tools. The unit of analysis was the individual teacher. Data were collected between January and May 2023 using an online questionnaire distributed through institutional contacts and professional teaching networks. A non-probability convenience sampling strategy was employed due to access constraints. While this approach limits statistical generalisability, it is commonly used in theory-driven educational research that examines relationships among latent constructs (Perez Rave et al., 2022). Therefore, the findings should be interpreted as explanatory rather than representative of all English teachers, consistent with theory-testing research designs. The implications of this sampling strategy are acknowledged in the Discussion section. Sample size adequacy was assessed using the gamma-exponential method proposed by Kock (2025). This method estimates the minimum required sample size for PLS-SEM based on statistical power, significance level, and the minimum expected path coefficient. Based on a power level of 0.80 and a significance level of 0.05, the minimum required sample size was 146. A total of 464 valid responses were obtained, substantially exceeding this requirement and supporting the robustness of the statistical analysis. The full collinearity variance inflation factor (FCVIF) was used in this work to analyse lateral collinearity. It is important to clarify that participation in the study did not require teachers to be active users of AI tools in writing instruction. Given the early stage of AI adoption at the time of data collection (2023), the sample may include teachers with varying levels of familiarity, ranging from no experience to exploratory or emerging use. The study, therefore, focuses on TPAI, IES, and IA related to AI-supported writing instruction rather than assuming uniform adoption. This approach is consistent with prior research examining technology integration readiness in early adoption phases.

The final dataset consisted of 464 valid responses from English language teachers working in formal educational institutions. Participants represented a wide range of teaching experience and institutional contexts. Teachers with 11–15 years of experience constituted the largest group, while university-level instructors represented approximately one quarter of the sample. This distribution indicates balanced representation across career stages and educational levels rather than dominance by any single group. To examine whether responses differed systematically across demographic categories, a one-way analysis of variance was conducted for teaching experience and institutional level. The results revealed no statistically significant differences (*p* > 0.05), suggesting that these background characteristics did not meaningfully shape perceptions and reported practices. Accordingly, all responses were retained for subsequent model testing. Given the early stage of AI adoption during the data collection period, participants likely represented different stages of familiarity and experimentation with AI tools, indicating heterogeneous levels of adoption rather than uniform implementation. The following Table 3 shows respondents’ demographic characteristics.

**Table 3:** Respondents’ demographic characteristics (*n* = 464).

Variable	Category	Frequency	Percentage
Teaching experience	0–5 years	104	22.4
	6–10 years	86	18.5
	11–15 years	127	27.4
	16–20 years	89	19.2
	More than 20 years	58	12.5
Institutional level	Secondary school	191	41.2
	College	162	34.9
	University	111	23.9
Prior exposure to AI tools	Low	146	31.5
	Moderate	198	42.7
	High	120	25.9

Source(s): authors own work.

### 3.6 Bias control and data consistency

Given the use of a cross-sectional, single-respondent survey design, potential CMB was addressed through both procedural and statistical measures. Procedurally, different Likert-scale formats and careful item wording were employed to reduce response pattern bias. Statistically, FCVIF was calculated following established procedures (Cheng et al., 2022). All values were below the recommended threshold, indicating that CMB was unlikely to pose a serious threat to the validity of the findings. All analyses were conducted using a single final dataset of 464 responses to ensure consistency across descriptive statistics, measurement model evaluation, and structural model testing. In addition, several procedural remedies recommended in survey research were implemented to reduce common method bias. These included ensuring respondent anonymity, conceptually separating construct measurement sections, and using different response scale formats. Together with the FCVIF results, these precautions suggest that common method bias is unlikely to affect the interpretation of the structural relationships substantially.

### 3.7 Mediation, moderation, and model evaluation procedures

Mediation and moderation effects were tested within the PLS-SEM framework using bootstrapping procedures with 5,000 resamples (Halis and Halis, 2022). Mediation was assessed by examining the significance of indirect effects. Moderation was tested by creating interaction terms between IA and IES. All indicators were mean-centred prior to creating interaction terms to reduce potential multicollinearity. Variance inflation factor values for all interaction models remained within acceptable limits. Model evaluation focused on the significance of path coefficients, the explained variance of endogenous constructs, and indicators of predictive relevance. These procedures ensured that the estimated relationships were statistically robust and theoretically interpretable.

### 3.8 Ethical considerations

Ethical principles were observed throughout the research process. Participation was voluntary, and respondents were informed about the purpose of the study, the anonymity of their responses, and their right to withdraw at any time. No personally identifiable information was collected. Formal institutional ethical approval was not required under departmental research guidelines; however, the study adhered to recognized ethical standards for educational research (Tatiana et al., 2025).

## 4 Research results

### 4.1 Preliminary model diagnostics and quality assessment

Prior to hypothesis testing, the structural model was evaluated for potential collinearity and common method bias, which are critical concerns in cross-sectional, single-respondent survey research (Castillo et al., 2025). In line with recommendations for partial least squares structural equation modeling, full collinearity variance inflation factor (FCVIF) values were employed to assess both lateral collinearity and common method bias simultaneously (Wang et al., 2024) The FCVIF results are reported in Table 4, where all construct-level values fall below the conservative threshold of 3.3, indicating that neither lateral collinearity nor common method bias poses a substantive threat to the validity of the model estimates (Merkle, 2025). This finding supports the suitability of the dataset for structural model evaluation. In addition to FCVIF, multicollinearity among formative indicators was examined using indicator-level variance inflation factors (VIFs), which are reported separately in Table 5. Table 5 reports the formative indicator weights, significance levels, loadings, and VIF values for all constructs, providing the main empirical basis for retaining the formative measurement specification. Most indicators exhibited VIF values below the strict cut-off of 3.3. Two indicators associated with pedagogical integration of AI-supported writing instruction slightly exceeded this threshold; however, their values remained below the more widely accepted upper limit of 5.0 for formative measurement models (Yu, 2025). Given the formative nature of the constructs and the theoretical relevance of these indicators, they were retained in the model, consistent with established methodological guidance. Model evaluation subsequently focused on explanatory power and predictive relevance rather than global goodness-of-fit indices, which are not considered decisive in PLS-SEM (Sharma et al., 2023). The model demonstrated adequate explanatory capability across all endogenous constructs, providing a robust foundation for hypothesis testing. All indicator weights were retained because they were either statistically significant or theoretically essential to preserving construct content coverage.

**Table 4:** Full collinearity variance inflation factor (FCVIF) results.

Study construct	FCVIF
Teachers’ perceptions of AI-supported writing instruction (TPAI)	2.38
Instructional agency (IA)	2.11
Professional development (PD)	2.46
Institutional and ethical support (IES)	2.29
Pedagogical integration of AI-supported writing instruction (PIAI)	2.63

Source(s): authors own work.

**Table 5:** Assessment of the formative measurement model.

Construct	Indicator	Weight	*p*-value	Indicator loading	VIF
Teachers’ perceptions of AI-supported writing instruction (TPAI)	TPAI1	0.264	0.041	0.821	2.04
	TPAI2	0.297	0.018	0.856	2.31
	TPAI3	0.281	0.027	0.844	2.22
	TPAI4	0.243	0.049	0.803	1.97
Instructional agency (IA)	IA1	0.231	0.046	0.762	1.88
	IA2	0.284	0.021	0.825	2.14
	IA3	0.198	0.033	0.741	1.93
	IA4	0.265	0.019	0.861	2.36
Professional development (PD)	PD1	0.318	0.002	0.872	2.67
	PD2	0.294	<0.001	0.889	2.91
	PD3	0.176	0.028	0.794	2.18
	PD4	0.159	0.034	0.768	2.05
Institutional and ethical support (IES)	IES1	0.219	0.039	0.773	2.09
	IES2	0.248	0.024	0.801	2.33
	IES3	0.271	0.015	0.836	2.58
	IES4	0.289	0.009	0.862	2.71
PIAI of AI-supported writing instruction (PIAI)	PIAI1	0.366	0.004	0.902	3.12
	PIAI2	0.214	0.026	0.781	2.44
	PIAI3	0.192	0.019	0.918	3.36
	PIAI4	0.173	0.011	0.934	3.58
	PIAI5	0.155	0.031	0.764	2.21

Source(s): authors own work.

### 4.2 Structural model evaluation

The explanatory power of the model was assessed using the coefficient of determination (R^2^), calculated as:


$$R^{2}=1-\frac{\sum_{i=1}^{n}{\left(y_{i}-{\hat{y}}_{i}\right)}^{2}}{\sum_{i=1}^{n}{\left(y_{i}-\overset{ˉ}{y}\right)}^{2}}$$

The model explained 16% of the variance in IA, 43% in PD, and 57% in the PIAI. The R^2^ value for PIAI indicates moderate explanatory power, consistent with benchmarks for behavioral and educational research (Hair et al., 2021). Predictive relevance was examined using the Stone–Geisser Q^2^ statistic obtained through blindfolding:


$$Q^{2}=1-\frac{\sum_{i=1}^{n}{\left(y_{i}-{\hat{y}}_{i}\right)}^{2}}{\sum_{i=1}^{n}{\left(y_{i}-\overset{ˉ}{y}\right)}^{2}}$$

The model also demonstrated satisfactory predictive relevance. Table 4 reports the full collinearity variance inflation factor (FCVIF) values for all study constructs. All FCVIF values were below the conservative threshold of 3.3, indicating that lateral collinearity and common method bias were not substantive concerns in this study (Aslam and Li, 2025).

**Figure 2:**
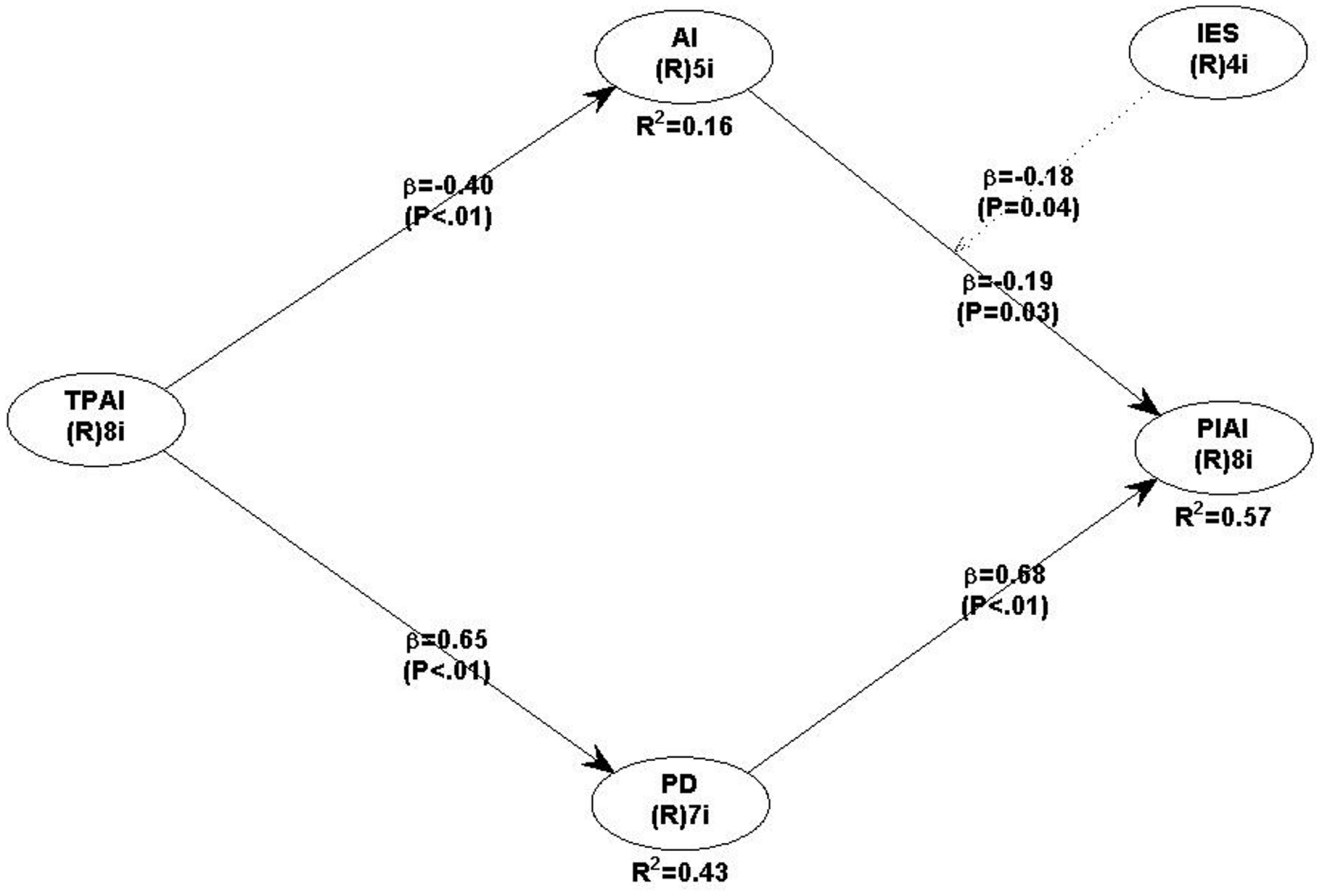
Structural model results. Source(s): authors own work. TPAI = Teachers’ perceptions of AI-supported writing instruction; IA = instructional agency; PD = professional development; IES = institutional and ethical support; PIAI = pedagogical integration of AI-supported writing instruction.

Following Kock and Dow (2025), FCVIF values were examined to assess the presence of CMB. All values were below the conservative threshold of 3.3, indicating that CMB was unlikely to affect the study’s findings. Figure 2 presents the results of the structural equation model estimated using PLS-SEM. The model illustrates the hypothesized relationships among TPAI, IA, PD, IES, and the PIAI. As shown in the figure, TPAI exert a significant positive effect on IA (*β* = 0.40, *p* < 0.01) and PD (*β* = 0.65, *p* < 0.01). IA (*β* = 0.19, *p* = 0.031) and PD (*β* = 0.68, *p* < 0.01) both contribute positively to PIAI, jointly explaining 57% of its variance (R^2^ = 0.57). In addition, IES significantly moderates the relationship between IA and PIAI (*β* = −0.18, *p* = 0.040). Overall, the model demonstrates satisfactory explanatory power, accounting for 16% of the variance in IA and 43% in PD, thereby providing empirical support for the proposed theoretical framework.

**Figure 3:**
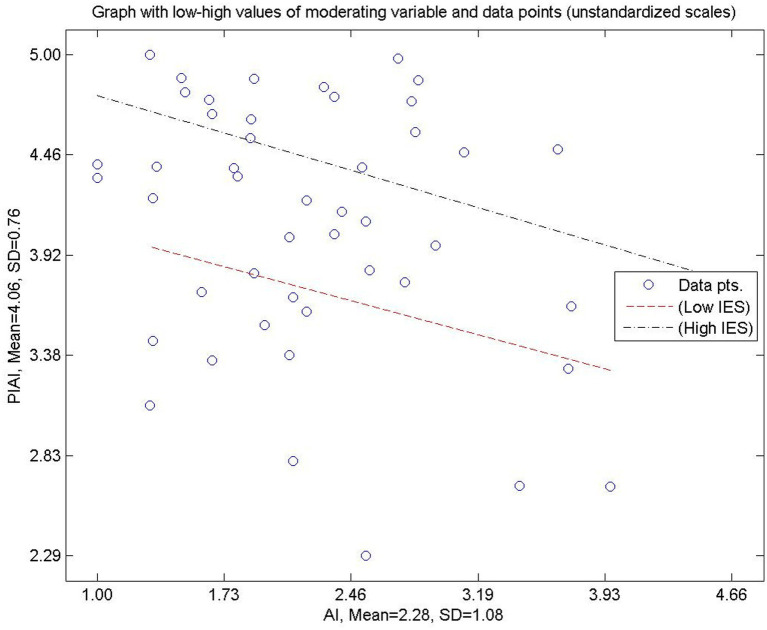
Linear graph displaying the impact of AI on PIAI across low and high degrees of IES. Source(s): authors own work.

Figure 3 depicts the linear moderating effect of IES on the relationship between IA and the PIAI. The figure plots simple slopes representing low and high levels of IES. The results indicate that IA is positively associated with PIAI at both levels of IES; however, the slope is steeper when IES is low. This pattern suggests that in contexts with weaker institutional guidance, PIAI relies more heavily on IA. Conversely, when IES is strong, the dependence of PIAI on IA is reduced, indicating a compensatory effect of organizational structures.

**Figure 4:**
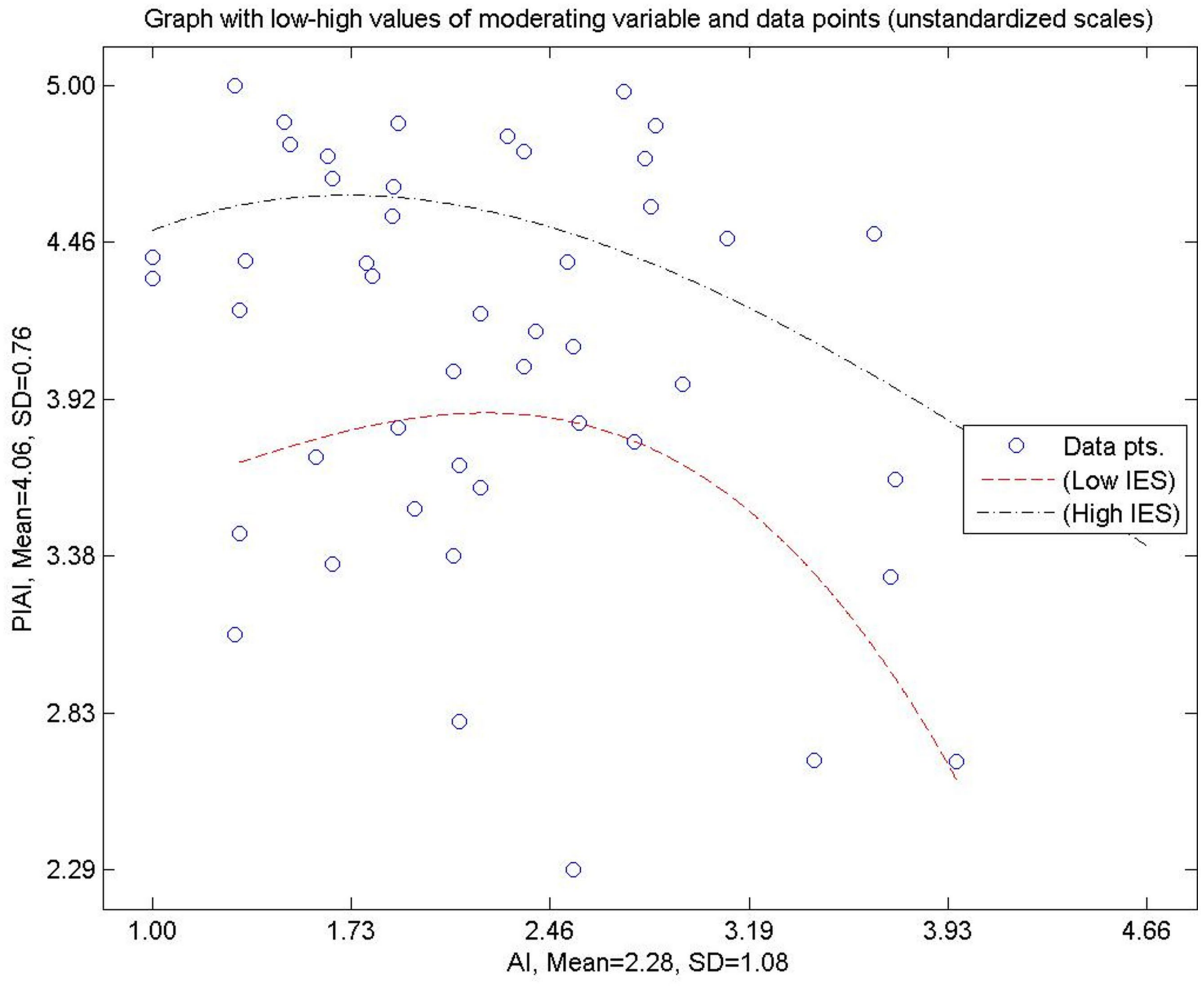
Warped relationship between AI and PIAI for both high and low levels of IES. Source(s): authors own work.

Figure 4 extends the linear analysis by illustrating the warped, non-linear relationship between IA and PIAI under conditions of low and high IES. The curved trajectories reveal that PIAI does not increase uniformly with IA. At lower levels of IES, integration initially rises with increasing IA but shows diminishing returns at higher levels. In contrast, under high IES, PIAI remains relatively stable across varying levels of IA. This non-linear pattern reinforces the interpretation that IES stabilizes instructional practice, reducing variability driven by IA.

**Figure 5:**
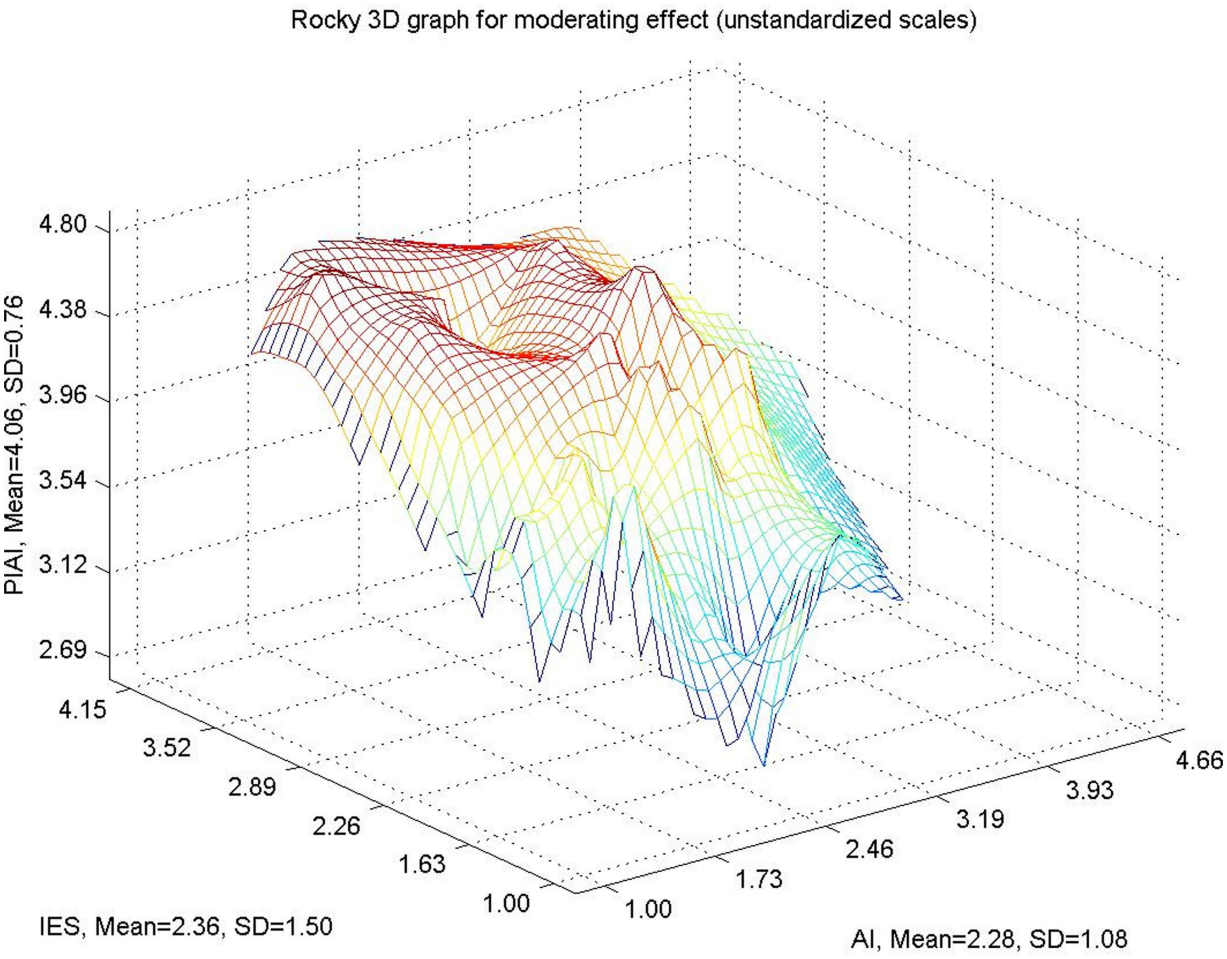
Three-dimensional interaction surface. Source(s): authors own work.

Figure 5 provides a three-dimensional visualization of the interaction between IA, IES, and PIAI. The surface plot demonstrates how PIAI varies simultaneously across different levels of IA and IES. Higher levels of IES are associated with more consistent and elevated levels of PIAI, even when IA is moderate. In contrast, when IES is low, PIAI fluctuates more sharply in response to changes in IA. This visualization complements the moderation analysis by offering a holistic representation of how individual and organizational factors jointly shape AI-supported writing practices.

### 4.3 Hypothesis testing

Hypotheses were tested using bootstrapping with 5,000 resamples. Table 6 summarizes the structural model results, including path coefficients, t-values, *p*-values, and effect sizes, to provide full transparency of the hypothesis testing results. All reported coefficients, significance levels, and effect sizes correspond exactly with Table 6 and Figure 2 to avoid numerical discrepancies. H1 proposed that TPAI positively influence IA. The results show a significant positive relationship (*β* = 0.40, *p* < 0.001), supporting H1. This indicates that favorable perceptions of AI tools are associated with stronger perceived capacity to exercise instructional judgement. H2 examined the relationship between IA and PIAI. IA exerted a positive and statistically significant effect on PIAI (*β* = 0.19, *p* = 0.031). Although the effect size is small, it is meaningful and supports H2, suggesting that the IA of teachers contributes to the PIAI in the classroom. H3 tested the mediating role of PD in the relationship between TPAI and IA. In addition, the model also examined the indirect contribution of PD to pedagogical integration (PIAI), reflecting the broader role of PD in translating teacher perceptions into instructional practice. Although the theoretical hypothesis focused on PD as a mediator between TPAI and IA, the empirical model also examined its indirect contribution to pedagogical integration, reflecting the broader role of PD in translating teacher perceptions into instructional practice.

**Table 6:** Structural model results.

Hypothesis	Structural relationship	Path coefficient (β)	t-value	*p*-value	Effect size (f^2^)	Decision
H1	TPAI → IA	0.40	5.94	<0.001	0.16	Supported
H2	IA → PIAI	0.19	2.17	0.031	0.04	Supported
H3	TPAI → PD → PIAI	0.44 (indirect)	4.12	<0.001	0.18	Supported
H4	IA × IES → PIAI	−0.18	2.05	0.040	0.03	Supported

Source(s): authors own work.

The indirect effect was statistically significant. The indirect coefficient was computed as the product of the relevant structural paths:


$$\beta_{\text{indirect}}=(\beta_{\text{TPAI}\to \mathrm{PD}})(\beta_{\mathrm{PD}\to \text{PIAI}})$$

$$\beta_{\text{indirect}}=(0.65)(0.68)=0.44$$

This result supports H3 and indicates that PD constitutes a substantial mechanism through which TPAIs are translated into pedagogical practice. H4 examined whether IES moderates the relationship between IA and PIAI. The interaction effect was negative and statistically significant (β = −0.18, *p* = 0.040), indicating a significant moderating effect. Although the direction differs from the expected positive moderation, the result still supports the presence of a moderation relationship, with IES operating as a compensatory rather than strengthening factor. The moderation model is expressed as:


$$Y=\beta_{0}+\beta_{1}X+\beta_{2}Z+\beta_{3}(X\times Z)+\varepsilon$$

Where Y represents PIAI, X represents IA, and Z represents IES. The negative coefficient suggests that when IES is strong, PIAI becomes less dependent on individual IA, reflecting the compensatory role of organisational guidance.

### 4.4 Effect size interpretation

Effect sizes were evaluated using Cohen’s $f^{2}$ :


$$f^{2}=\frac{R_{\text{included}}^{2}-R_{\text{excluded}}^{2}}{1-R_{\text{included}}^{2}}$$

Effect size interpretation followed conventional thresholds of 0.02 (small), 0.15 (medium), and 0.35 (large). The mediating effect involving PD demonstrated a medium practical contribution, while the moderation effect exhibited a small but meaningful influence. These results are interpreted conservatively to avoid overstating practical impact.

### 4.5 Consistency and clarity check

All numerical values reported in this section correspond directly to Table 6 and Figure 2. No discrepancies were identified between coefficients, R^2^ values, or significance levels across text, tables, and figures. Each hypothesis is explicitly linked to its supporting statistical evidence, providing a clear quantitative foundation for the discussion that follows.

## 5 Discussion

This study set out to explain how English TPAI translate into pedagogical practice by examining the intervening roles of IA, PD, and IES. Rather than treating AI integration as a linear adoption process, the findings highlight the importance of teacher-centred mechanisms and contextual conditions that shape instructional decision-making. Interpreted conceptually, the results demonstrate that the PIAI emerges through the interaction between individual professional capacity and organizational structures. This finding reinforces the view that IA operates within institutional conditions rather than independently of them.

### 5.1 Teachers’ perceptions of AI-supported writing instruction (TPAI) and instructional agency (IA)

The positive association between TPAI and IA supports theoretical perspectives that position teacher cognition as a foundational driver of instructional change. This result was expected and aligns with research showing that teachers’ beliefs strongly influence how innovations are interpreted and enacted in practice. When AI tools are perceived as pedagogically legitimate and aligned with writing objectives, teachers appear more willing to exercise autonomy in instructional planning and classroom experimentation. This finding reinforces IA-based theories of teaching, which emphasize that professional action is shaped not by technology itself, but by teachers’ meaning-making processes. Compared with studies conducted in stable market education systems, where institutional frameworks often normalize digital innovation, the present findings suggest that perception plays a more critical role in contexts characterized by policy ambiguity and uneven guidance. In such environments, teachers rely more heavily on personal judgment to determine whether and how to integrate AI, amplifying the influence of individual perceptions on IA.

### 5.2 Instructional agency (IA) and pedagogical integration of AI-supported writing instruction (PIAI)

The significant relationship between IA and PIAI confirms that IA functions as a key mechanism linking belief to practice. Teachers who report higher IA are better positioned to embed AI tools into writing instruction in pedagogically meaningful ways, such as supporting feedback, revision, and individualized learning. This finding is consistent with sociocultural models of teaching that conceptualize instruction as an adaptive practice shaped by professional discretion rather than procedural compliance. Importantly, the result extends prior literature by showing that IA is not merely an outcome of IES, but an active contributor to instructional innovation. In contrast to research on highly regulated systems, where standardized policies often guide technology use, the current findings suggest that IA becomes especially salient when teachers must navigate uncertainty about assessment norms, academic integrity, and ethical boundaries related to AI use.

### 5.3 Direct effects of TPAI on pedagogical integration of AI-supported writing instruction (PIAI)

The direct relationship between TPAI and PIAI indicates that favorable beliefs about AI can, in some cases, translate directly into classroom practice. However, the modest strength of this relationship suggests that perception alone is insufficient to sustain high-quality integration. This partially diverges from findings in stable market economies, where positive attitudes toward educational technology more consistently predict classroom use due to stronger infrastructural and policy alignment. Conceptually, this divergence can be explained by the absence of fully institutionalized norms for AI-supported writing instruction. Without clear guidance on acceptable use, assessment alignment, and ethical boundaries, teachers may hesitate to operationalize positive perceptions into consistent pedagogical routines. This insight contributes theoretically by highlighting the conditional nature of perception-based models of technology adoption in emerging instructional domains. This suggests that PD may function both as a mechanism supporting teachers’ IA and as a practical enabler of pedagogical AI integration.

### 5.4 Moderating role of institutional and ethical support (IES)

Although the hypothesis predicted a positive moderating effect, the observed interaction coefficient was negative, indicating that IES operates as a compensatory rather than amplifying factor. IES were found to moderate the relationship between IA and PIAI, although the effect was relatively weak. Rather than indicating limited relevance, this pattern suggests that IES functions as a stabilizing mechanism rather than a primary driver of innovation. Clear ethical guidelines and institutional endorsement appear to reduce perceived risk and legitimize experimentation, allowing teachers to act on their IA with greater confidence. The weak moderation effect may reflect the transitional stage of AI governance in education. In contrast to stable market contexts where institutional frameworks strongly shape instructional practice, AI-related policies remain emergent and unevenly implemented. As a result, teachers continue to rely primarily on professional judgment and informal learning networks. From a theoretical perspective, this finding implies that resilience and instructional innovation under uncertainty are driven more by individual adaptive capacity than by formal structures, at least in the early phases of technological diffusion. This directional difference suggests that strong IES may reduce reliance on individual IA rather than strengthening its effect.

### 5.5 Reintegrating the shared value framework

Viewed through a shared value framework, the findings suggest that AI-supported writing instruction generates educational value only when individual and institutional logics are aligned. TPAI and IA contribute to value creation at the classroom level by shaping meaningful pedagogical use. IES, meanwhile, plays a protective role by preventing potential disvalue such as misuse, inequity, or erosion of professional norms. The relatively weak moderating effect indicates that shared value in AI-supported instruction is still developing rather than fully embedded. Value creation currently depends more on individual professional capacity than on organizational systems, highlighting a gap between technological potential and institutional readiness. This theoretical contribution extends shared value thinking to educational technology by demonstrating that value and disvalue coexist during periods of innovation, particularly when governance structures lag behind practice.

### 5.6 Integrated theoretical and practical implications

Taken together, the findings advance a teacher-centred model of AI-supported writing instruction that integrates TPAI, IA, PD, and IES context. Theoretically, the study moves beyond adoption-focused explanations by clarifying the mechanisms through which AI becomes pedagogically meaningful. Practically, the results suggest that PD should prioritize instructional design, ethical reasoning, and opportunities for reflective practice rather than focusing solely on technical training. At the institutional level, flexible yet explicit ethical frameworks are needed to support innovation while preserving professional autonomy. Overall, the discussion demonstrates that dynamic interactions between IA and organizational conditions shape effective AI integration in writing instruction. Rather than being determined by the availability of technology, PIAI emerges through negotiated processes of meaning-making, professional judgment, and IES. This finding is particularly important in writing instruction because AI tools may directly influence drafting, feedback, and revision processes, which require careful pedagogical judgement from teachers. This study makes several contributions to research on AI-supported writing instruction. First, it integrates TPAI, IA, PD, and IES into a single empirical framework, which has rarely been examined together in prior studies. Second, the study contributes to writing education research by examining AI integration from a teacher professional decision-making perspective rather than focusing only on technological tools or student outcomes. Third, by examining both individual and contextual factors simultaneously, the findings provide insight into how early adoption of AI in writing instruction may be shaped by the interaction between teacher preparedness and institutional conditions. While the study does not examine specific PD models or institutional policies, it provides empirical evidence about key factors associated with pedagogical AI integration in writing instruction.

## 6 Conclusion

This study examined how English TPAI influence the PIAI through IA, PD, and IES. By adopting a teacher-centred analytical lens, the research moves beyond technology-centric explanations of AI adoption. It demonstrates that meaningful instructional integration depends on the interaction between individual cognition, professional capacity, and organizational context. The findings indicate that AI-supported writing instruction becomes pedagogically effective not simply through access to tools, but through teachers’ capacity to interpret, adapt, and legitimize their use within established instructional and ethical frameworks. Overall, the results provide empirical support for a multilevel explanatory model of AI-supported writing instruction, highlighting the importance of aligning teacher beliefs, professional learning opportunities, and institutional guidance to realize AI’s instructional potential in language education.

### 6.1 Theoretical contributions

This study makes several theoretical contributions to the literature on artificial intelligence in education and teacher cognition. First, it extends teacher perception and agency theories by empirically demonstrating that TPAI serve as a foundational mechanism that activates IA. This finding reinforces the view that IA is not an inherent attribute, but an emergent capacity shaped by teachers’ interpretations of instructional tools and pedagogical value. Second, the study advances PD theory by positioning PD as an explanatory pathway rather than a contextual background variable. The results show that PD plays a central role in translating favorable perceptions of AI into sustained PIAI, thereby clarifying how instructional innovation is operationalized in classroom practice. Third, by incorporating IES as a moderating condition, the research contributes to emerging scholarship on AI governance in education. The findings suggest that institutional structures shape the enactment of IA of teachers, particularly in contexts where ethical uncertainty and policy ambiguity surround AI use. Finally, by integrating these elements within a single empirical model, the study contributes a teacher-centred theoretical framework that explains not only whether AI is adopted, but how and under what conditions it becomes pedagogically meaningful.

### 6.2 Practical contributions

From a practical perspective, the findings offer important implications for teachers, educational leaders, and policymakers. For practitioners, the results highlight the need to move beyond surface-level adoption of AI tools and focus instead on pedagogical design, reflective practice, and instructional decision-making. The findings suggest that PD may play an important role in supporting teachers’ pedagogical integration of AI in writing instruction. Although the study did not examine specific PD program designs, the results indicate that professional learning opportunities related to instructional application may be important for supporting AI integration. For educational leaders, the findings underscore the importance of establishing clear but flexible institutional and ethical frameworks that support innovation while safeguarding professional standards. The results also highlight the potential importance of institutional and ethical guidance in supporting teachers’ instructional decision-making related to AI use. While this study did not directly measure teachers’ confidence, the findings suggest that supportive institutional conditions may help create environments where teachers can exercise their IA more effectively. At the policy level, the study suggests that investments in AI infrastructure should be accompanied by sustained support for teacher learning and organizational capacity-building to ensure that AI integration contributes positively to writing instruction. More specifically, the findings suggest practical relevance for writing instruction because AI tools may directly influence key writing processes such as drafting, feedback, and revision. From a pedagogical perspective, this highlights the importance of supporting teachers in making informed instructional decisions about when AI assistance should support learning and when independent writing practice should be emphasized. The results therefore suggest that AI integration in writing instruction should be understood not only as technology adoption but as part of broader instructional decision-making related to writing development.

### 6.3 Limitations and further research

Despite its contributions, this study has limitations that warrant consideration. First, the research employed a cross-sectional, questionnaire-based design, which limits causal interpretation and captures TPAI at a single point in time. Future research could adopt longitudinal designs to examine how perceptions, agency, and pedagogical integration evolve as AI technologies and institutional policies mature. Second, the study relied on self-reported data, which may be subject to response bias. Subsequent studies could incorporate classroom observations, instructional artefacts, or student learning outcomes to triangulate findings and strengthen empirical validity. Third, although the sample included teachers from different institutional levels, the findings may not fully reflect variations across national education systems or policy environments. Comparative research across countries and educational contexts would deepen understanding of how institutional conditions shape AI-supported writing instruction. Future research could also explore additional mediating or moderating factors, such as assessment practices, curriculum constraints, or peer collaboration, to further refine the proposed model. Such extensions would enhance theoretical precision and support the development of more context-sensitive strategies for AI integration in language education. An additional limitation concerns the timing of data collection. The survey was conducted in 2023, when AI adoption in education was still developing and levels of teacher experience varied considerably. As a result, responses may reflect perceived readiness and early experimentation rather than mature classroom integration. Future studies could distinguish more explicitly between non-users, novice users, and experienced adopters to further strengthen interpretation of AI integration processes. Future research could further distinguish the different pathways through which PD influences both IA and pedagogical integration. Several methodological limitations should be considered when interpreting the findings. First, the study used convenience sampling, which may limit the generalizability of the results beyond the study context. Second, the cross-sectional design captures TPAI and reported practices at one point in time and therefore does not allow causal conclusions about how AI integration develops over time. Third, the study relies on self-reported data, which may be influenced by social desirability or differences in teachers’ interpretations of AI use. Fourth, because data were collected in 2023 during the early stages of AI adoption in education, teachers may have differed in their familiarity and practical experience with AI tools. Future research could address these limitations through longitudinal designs, mixed-methods approaches, and comparisons across different educational contexts. Despite these limitations, the study provides useful insights into early patterns of teacher readiness and pedagogical decision-making related to AI-supported writing instruction.

## Data Availability

The original contributions presented in the study are included in the article/supplementary material, further inquiries can be directed to the corresponding author.
